# MC3T3 osteoblast-like cells cultured at alkaline pH: Microarray data (Affymetrix GeneChip Mouse 2.0 ST)

**DOI:** 10.1016/j.dib.2017.05.013

**Published:** 2017-05-10

**Authors:** Anne-Marie Galow, Alexander Rebl, Dirk Koczan, Jan Gimsa

**Affiliations:** a University of Rostock, Gertrudenstr. 11a, 18057 Rostock, Germany; b Institute of Genome Biology, Leibniz Institute for Farm Animal Biology, Wilhelm-Stahl-Allee 2, 18196 Dummerstorf, Germany; c Institute of Immunology, University of Rostock, Schillingallee 70, 18055 Rostock, Germany

**Keywords:** Bone, Gene regulation, Functional gene networks, p38 MAPK, Murine cell line, Quantitative real-time PCR, Robust Multi-Array Average, Fold change bias, Differential gene expression, Ingenuity Pathway Analyses

## Abstract

It is well known that pH plays a pivotal role in the control of bone remodeling. However, no comprehensive gene expression data are available for the effects of alkaline pH on osteoblasts. We cultured differentiating MC3T3-E1 osteoblast-like cells at pH 7.4, 7.8, and 8.4 for 14 days. To identify differential gene expression, microarray data were collected with Affymetrix GeneChips. The data were validated by real-time PCRs for five genes that were found to be greatly regulated in the GeneChip-experiments (DMP1, FABP4, SFRP2 and TNFRSF19) or considered relevant for the terminal function of osteoblasts (DMP1 and ATF4). All the data are available from the Gene Expression Omnibus database (GEO accession: GSE84907). Here, we provide pathway analyses of known protein coding genes that were down-regulated or up-regulated by greater than 2.0-fold. The regulation datasets obtained from comparisons of pH 7.8 and 7.4, as well as pH 8.4 and 7.4 share a high number of differentially expressed genes. When comparing pH 8.4 and 7.8, other genes mainly emerge, suggesting not only a simple amplification of the effects at pH 8.4 that were already induced at pH 7.8 but also the induction of different pathways. For a more detailed analysis, different mammalian functional gene networks were assigned to each dataset. After merging and manual optimization of the network graphs, three combined functional gene networks were obtained that reflected distinct pH-dependent cellular responses. A common feature of the networks was the central role of p38 MAP kinase. The microarray data presented here are related to the research article doi:10.1016/j.bbrep.2017.02.001 (Galow et al., 2017) [1].

## **Specifications Table**

**Table:** 

Subject area	*Cell biology*
More specific subject area	*Bone cell biology, gene regulation*
Type of data	*Tables, schematic images*
How data was acquired	*Affymetrix GeneChip, Mouse Gene 2.0 ST Array, Robust Multi-Array Average and statistical analyses*
Data format	*Raw, filtered, analyzed*
Experimental factors	*Cells were cultured in HEPES buffered α-MEM at pH 7.4 (control) and under alkaline conditions at pH 7.8 and pH 8.4 for 14 days*
Experimental features	*RNA isolation, global gene expression analyses*
Data source location	*Rostock, Germany*
Data accessibility	*Pathway analyses data are within this article; microarray data are available from the Gene Expression Omnibus database (GEO accession:*GSE84907*)*

## **Value of the data**

• A benchmark global analysis of differential gene expression of osteoblast-like cells cultured under standard and alkaline pH conditions.
• These data may be useful for comparison with microarray data from other osteoblast cell lines or primary cells under different pH conditions.
• The data can serve as a reference for scrutinizing the culture conditions used for different cell types.
• Genes and pathways identified as differentially expressed in this data set could be investigated in future studies of the cell and molecular biology of bone regeneration.

## 1 Data

**Fig. 1:**
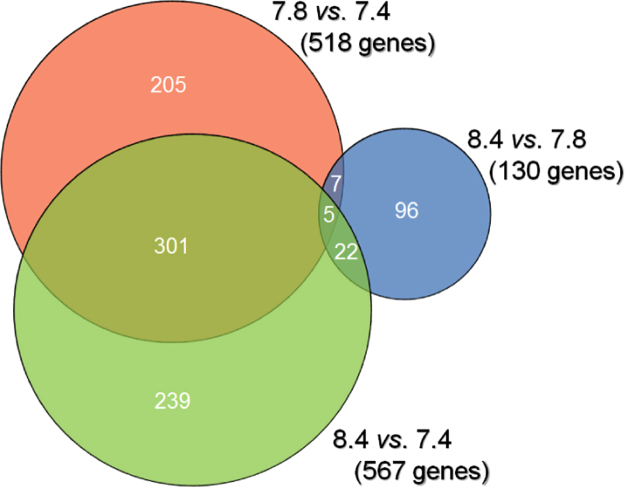
Venn diagram illustrating the numbers of genes, which were commonly and exclusively expressed at pH 7.8 and 7.4 (red circle), at pH 8.4 and 7.4 (green), as well as at pH 8.4 and 7.8 (blue).

Affymetrix GeneChip microarray analyses comparing mRNA isolated from MC3T3 osteoblast-like cells cultured at different pH revealed several hundred genes to be differentially regulated at pH 7.8 and pH 8.4 compared to the standard conditions (pH 7.4). Of more than 30,000 genes per chip, a list of >800 genes that were differentially expressed by greater than 2.0-fold (*p*<0.05) was generated [2], containing more than 500 differentially expressed (DE) genes for the comparison with pH 7.8 and approximately 550 for the comparison with pH 8.4. For the comparison of pH 8.4 with 7.8, the DE gene list contained approximately 130 genes. The comparison of the microarray datasets clearly showed only five DE genes that were common to each of the three pairwise comparisons. Other DE genes were present in one of the three datasets only, 239 genes in the pH 8.4 and 7.4 comparison, 205 genes in the pH 7.8 and 7.4 comparison, and 96 genes in the pH 8.4 and 7.8 comparison. Fig. 1 presents the relationships. Starting from the DE gene list, three functional gene networks were generated, using the IPA (Ingenuity Pathway Analyses; www.qiagenbioinformatics.com) software. The networks demonstrated distinct pH-dependent cellular responses. A common feature of each of the networks was the central role of p38 MAP kinase.

## 2 Experimental design, materials and methods

### 2.1 Cell culture

The mouse-osteoblast precursor cell line MC3T3-E1 [3] was obtained from the German collection of microorganisms and cell culture (DSMZ, Braunschweig, Germany) and cultured at pH 7.4, 7.8 or 8.4 as described previously [1].

### 2.2 Array hybridization and data validation

For gene expression analysis 10 mg/ml beta-glycerol phosphate and 10 ng/ml calcitriol were added after seven days to restrict the proliferation and support the differentiation of the cells. After 14 days, RNA isolation, RNA quantification and array hybridization were performed as described previously [1].

To validate the GeneChip data, quantitative real-time PCRs (qRT-PCRs) were performed for five genes that were found to be greatly regulated in the GeneChip experiments (DMP1, FABP4, SFRP2 and TNFRSF19) or considered relevant for the terminal function of osteoblasts (DMP1 and ATF4). After the RNA isolation described before [1], the RNA was adjusted to 800 ng per 20 µl sample. Reverse transcription reactions were run using the High Capacity cDNA Reverse Transcription Kit (Applied Biosystems™, Waltham, MA, USA) according to the manufacturer׳s protocol.

Real-time PCR was performed with total PCR mixture volumes of 20 µl, including 1 µl cDNA in a 7900HT Fast Real-Time PCR System (Applied Biosystems, Foster City, CA, USA) using the TaqMan™ Universal PCR Master Mix (Applied Biosystems™, Waltham, MA, USA) and predesigned primers and probes (DMP1 Mm01208363_m1, FABP4 Mm00445878_m1, SFRP2 Mm01213947_m1, TNFRSF19 Mm00443506_m1, ATF4 Mm00515324_m1). Glyceraldehyde-3-phosphate dehydrogenase (GAPDH; Applied Biosystems™, Waltham, MA, USA) was chosen as the reference gene. All samples were run in triplicate, and the mRNA contents of each target gene were normalized to GAPDH. The results were analyzed using the relative expression software tool REST-384 (http://rest.gene-quantification.info/).

The exemplary qRT-PCR results were largely consistent with the respective GeneChip data (see Supplementary file 1) . The systematic differences in the fold change determination between the two methods were caused by the limited dynamic range of the microarray technology compared with qRT-PCRs. This bias is increased by the model-based probe level analysis algorithm ׳Robust Multi-Array Average׳ used, which underestimates the fold change numbers of microarrays relative to qRT-PCR numbers [4], [5].

### 2.3 Statistical analyses of changes in global gene expression

Primary data analysis was carried out with the Affymetrix Expression Console 1.4.1.46 software including the Robust Multiarray Average module for normalization. Gene expression data were log-transformed. A change was considered significant when the false discovery rate-corrected p-value/q-value thresholds met the criterion *q*<0.01 at fold changes >|2|, i.e. expression increments or declines larger than two. A general overview of the DE gene sets at alkaline pH is presented in the Venn diagram [6] of Fig. 1.

### 2.4 Network analyses

We extracted functional networks corresponding to those known from mammalian (mouse, rat or human) *in vivo* and *in vitro* systems. For this, lists of DE genes were imported into the IPA software. The ‘Core Analysis’ routine of the software returned a list of networks with descending scores (see Supplementary file 2). The network scores are based on the hypergeometric distribution and are calculated with the right-tailed Fisher׳s Exact Test. For each pH comparison, the three networks with the highest scores were merged and manually optimized (Fig. 2).

**Fig. 2:**
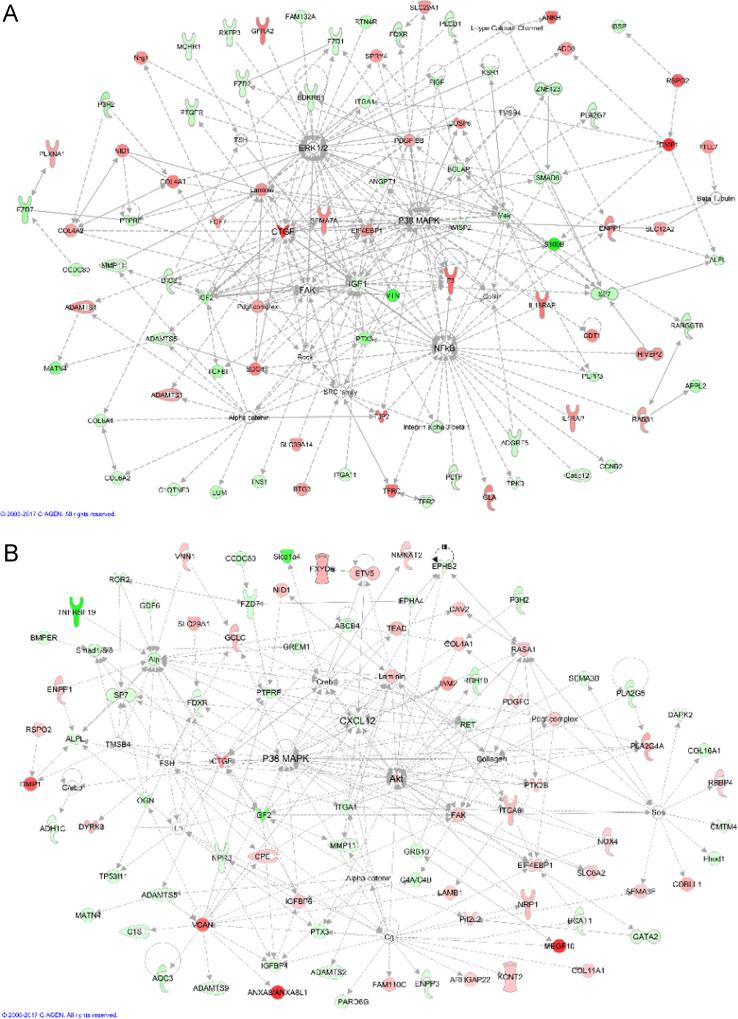
The network analyses illustrate the interactions of genes and gene products that are differentially regulated in MC3T3 osteoblast-like cells, which were cultured at different pH. The networks A (pH 7.8 vs. 7.4), B (pH 8.4 vs. 7.4), and C (pH 8.4 vs. 7.8) were obtained after manual optimization by combining the three functional networks given in Table 1 for each dataset. Red and green symbols represent up- and downregulated genes, while white symbols represent other compounds, which are required for the consistency of the networks, such as hormones, transcription factors, cytokines, receptors etc. Direct and indirect relationships are indicated by full and broken lines, respectively. Blocked lines indicate inhibiting effects.

**Fig. 2:**
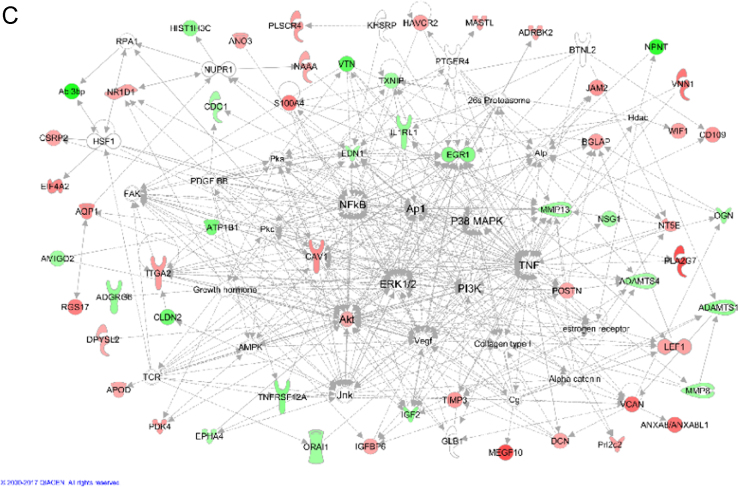
Continued.

Nevertheless, greatly regulated genes may be missing, because they do not emerge in the functional contexts of the networks chosen, like for example the SFRP2 gene. Please compare to Table 3 in [1]. Out of the 10 greatly regulated genes listed in this table, only five emerged in the networks in Fig. 2. The networks for each dataset are listed in Table 1.

**Table 1:** Designations of functional networks of "top diseases and functions" known from mammalian (mouse, rat or human) *in vivo* and *in vitro* systems, which were extracted by the IPA software using the datasets obtained from the different pH comparisons. For each pH comparison, the table presents the three functional networks with the highest scores and the number of DE genes involved in the networks.

**Dataset**	**Score**	**Genes involved**	**Functional networks (top diseases and functions)**
**pH 7.8 vs. 7.4**	40	29	Cell morphology, cellular assembly and organization, cellular function and maintenance
	40	29	Connective tissue disorders, inflammatory disease, inflammatory response
	36	27	Cellular development, cellular growth and proliferation, nervous system development and function
**pH 8.4 vs. 7.4**	39	29	Embryonic development, organ development, organ morphology
	37	28	Connective tissue disorders, cellular assembly and organization, cellular function and maintenance
	35	27	Connective tissue development and function, skeletal and muscular system development and function, tissue development
**pH 8.4 vs. 7.8**	43	23	Cellular development, cellular growth and proliferation, organ development
	38	21	Molecular transport, cardiovascular system development and function, organismal development
	24	15	Skeletal and muscular system development and function, carbohydrate metabolism, nucleic acid metabolism

## Footnotes

Appendix A Supplementary data associated with this article can be found in the online version at http://dx.doi.org/10.1016/j.dib.2017.05.013.
